# Causal inference can lead us to modifiable mechanisms and informative archetypes in sepsis

**DOI:** 10.1007/s00134-024-07665-4

**Published:** 2024-10-21

**Authors:** J. Kenneth Baillie, Derek Angus, Katie Burnham, Thierry Calandra, Carolyn Calfee, Alex Gutteridge, Nir Hacohen, Purvesh Khatri, Raymond Langley, Avi Ma’ayan, John Marshall, David Maslove, Hallie Prescott, Kathy Rowan, Brendon Scicluna, Christopher Seymour, Manu Shankar-Hari, Nathan Shapiro, W. Joost Wiersinga, Mervyn Singer, Adrienne G. Randolph

**Affiliations:** 1 Baillie Gifford Pandemic Science Hub, Centre for Inflammation Research, The Queen’s Medical Research Institute, University of Edinburgh, 47 Little France Crescent, Edinburgh, UK; 2 Roslin Institute, University of Edinburgh, Easter Bush, Edinburgh, EH25 9RG, UK; 3 MRC Human Genetics Unit, Institute of Genetics and Cancer, University of Edinburgh, Western General Hospital, Crewe Road, Edinburgh, EH4 2XU, UK; 4 Intensive Care Unit, Royal Infirmary of Edinburgh, 54 Little France Drive, Edinburgh, EH16 5SA, UK; 5 International Sepsis Forum; 6 Department of Critical Care Medicine, University of Pittsburgh; 7 UPMC Health System, Pittsburgh, Pennsylvania; 8 Wellcome Sanger Institute, Hinxton, UK; 9 Centre Hospitalier Universitaire Vaudois, Lausanne, Switzerland; 10 Division of Pulmonary, Critical Care, Allergy and Sleep Medicine, Departments of Medicine and Anesthesia, University of California San Francisco, San Francisco, CA, USA; 11 Computational Biology, GSK, Stevenage, UK; 12 Harvard University, Boston, USA; 13 Research Institute for Immunity, transplantation and infection, 240 Pasteur Dr Rm 1553, Biomedical Innovation Building, Palo Alto, California, USA; 14 College of Medicine, University of New Mexico, Albuquerque, New Mexico, United States; 15 Icahn School of Medicine at Mount Sinai, New York, NY; 16 St Michael’s Hospital, Toronto, ON M5B 1W8, Canada; 17 Department of Critical Care Medicine, Queen’s University, Kingston, Ontario, Canada; 18 University of Michigan, Michigan, USA; 19 Intensive Care National Audit & Research Centre, London, UK; 20 University of Malta, Malta; 21 Centre for Inflammation Research, The Queen’s Medical Research Institute, University of Edinburgh, 47 Little France Crescent, Edinburgh, UK; 22 Division of Infectious Diseases, Amsterdam UMC, University of Amsterdam, Netherlands; 23 University College London, London, UK; 24 Department of Anesthesiology, Critical Care and Pain Medicine, Boston Children’s Hospital, Boston, MA, USA; 25 Departments of Anaesthesia and Pediatrics, Harvard Medical School, Boston, MA, USA

## Abstract

**Purpose:**

Medical progress is reflected in the advance from broad clinical syndromes to mechanistically-coherent diagnoses. By this metric, research in sepsis is far behind other areas of medicine - the word itself conflates multiple different disease mechanisms, whilst excluding noninfectious syndromes (e.g. trauma, pancreatitis) with similar pathogenesis. New technologies, both for deep phenotyping and data analysis, offer the capability to define biological states with extreme depth. Progress is limited by a fundamental problem: observed groupings of patients lacking shared causal mechanisms are very poor predictors of response to treatment.

**Results:**

Here we discuss concrete steps to identify groups of patients reflecting archetypes of disease with shared underlying mechanisms of pathogenesis. Recent evidence demonstrates the role of causal inference from host genetics and randomised clinical trials to inform stratification analyses. Genetic studies can directly illuminate drug targets, but in addition they create a reservoir of statistical power that can be divided many times among potential patient subgroups to test for mechanistic coherence, accelerating discovery of modifiable mechanisms for testing in trials. Novel approaches, such as subgroup identification in-flight in clinical trials, will improve efficiency.

**Conculsion:**

Within the next decade, we expect ongoing large-scale collaborative projects to discover and test therapeutically-relevant sepsis archetypes.

## Introduction

### The sepsis hypothesis and a half-century of disappointment

While other areas of medicine have progressed into the mechanistic era, critical care in 2024 remains a speciality of syndromes, rather than diseases. Here, we build on our previous work on theoretical foundations^1^ and potential implications^2^ of subgrouping in critical illness, to describe practical steps for future research. This report describes a consensus among the authors, built over several years of discussion.

A 2014 review found that more than 120 published large-scale clinical trials of treatments for sepsis have been conducted,^3^ each costing millions of dollars (current estimates of Phase II/III trial costs range from $7-50 million^4^). None of these trials has yielded an effective new treatment. Sepsis is still considered an “orphan” disease with no approved therapies, with the lone exception of severe COVID-19. Since sepsis lumps together patients with fundamentally different disease pathogenesis,^5^ it is likely that some effective therapies have been rejected that could succeed in the right subgroup.^6^ The problem may be particularly difficult in sepsis compared to other syndromes: the biological processes triggered in sepsis have evolved to become extremely complex, making the effect of new treatments very difficult to predict.^7^

Significant progress has been made in recent years in recognising the heterogeneity of patients with sepsis, summarised in our previous work.^1,2^ The development of new technologies - here, together referred to as “deep phenotyping” - enables us to make millions of observations about a single patient with sepsis. This refers both to high-throughput biological read-outs in genomics and transcriptomics, and to automated collation of information from clinical records, monitoring, imaging and other routine tests.

Numerous studies demonstrate that deep phenotyping reveals features of critically ill patients that are not clinically obvious.^8–15^ If the host response to sepsis is treatable, then it is reasonable to expect that some of these observations are either biomarkers for, or direct measurements of, underlying processes that can be modified to prevent death.^16^ The distinguishing feature of these *modifiable* mechanisms is that they have a causal relationship with clinical outcomes.

Where the number of observations greatly exceeds the number of patients, there is a high probability that important signals will be present but cannot be detected with any confidence, because there is too much random noise. Secondly, since biology is reliably more complex than our understanding of it, causal relationships in high-dimensional biological data are difficult to distinguish from correlations. These fundamental limitations have probably prevented discovery of mechanistically-coherent diagnoses within sepsis.

The 1992 systemic inflammatory response syndrome (SIRS) concept^17^ summarised the prevailing clinical perception that immune processes associated with organ failure extend across infections and sterile injuries. This was proposed at around the same time as the prescient reasoning by Janeway that antibodies and T-cell receptors could not explain the speed of immune responses to pathogens, and hence that a broader, more ancient recognition system must exist.^18^ Subsequent research has revealed that innate immune systems across the tree of life possess a range of pattern recognition receptors^19^ that respond not only to molecular patterns found on infectious organisms, but also to similar patterns released by damaged cells^20^ (danger-associated molecular patterns, DAMPs),^21^ by initiating non-specific immune responses that may cause organ damage. The clinical concept of systemic inflammatory response might be described in molecular biological terms as DAMP-osis. Perhaps if SIRS had been proposed as a clinical observation, or a theoretical concept,^22^ rather than a clinical syndrome with the expectation of specific therapeutic responses, it might have better stood the test of time.^23^

A syndrome definition can err by inclusion and by exclusion. The definition of sepsis^24^ makes both errors: it is simultaneously too broad and too narrow. A single syndrome that combines coma due to meningitis, respiratory failure due to critical Covid-19, and cardiovascular shock due to urosepsis is self-evidently incoherent: it is very likely that distinct causal mechanisms underlie outcomes in these diseases. In part as a consequence, it also errs by exclusion - although classifiers exist,^25^ to our knowledge no immune process has been discovered to be shared across the whole spectrum of patients with sepsis, that is not also evident in patients with severe systemic inflammation precipitated by sterile injury such as trauma, pancreatitis, haemorrhage and burns.

Critical care medicine does not need a new definition of sepsis. Instead, we anticipate that future progress will ultimately replace the sepsis syndrome with overlapping, mechanistically-coherent diagnoses with specific therapeutic consequences. Here, we report a series of recommendations following an ongoing discussion among an international group of experts across a range of relevant disciplines,^1,2^ and consider some concrete steps that can be taken to discover and modify causal mechanisms of disease in sepsis.

**Definitions**

**Endotype**: A subgroup within a population of patients who are distinguished by a shared disease process.^26^ Historically, the underlying mechanism is what distinguishes a disease from a syndrome.

Trait*: A specific distinguishing characteristic of an organism.

Phenotype*: An observable characteristic of an organism.

Genotype*: The genetic makeup of an organism

**Treatable trait**: The pathophysiological feature (or, in a looser sense, a biomarker or group of biomarkers for that feature) that determines whether a given therapy will improve a given patient’s outcome. The same trait may be present in many different clinical syndromes or disease processes.^26^

**Modifiable mechanism**: An underlying disease process that can be modified by treatment.

**Archetype**: A description of the idealised, typical appearance representative of a group.

* these words are used widely in biology under these, or similar, definitions, so it would be undesirable for clinical researchers to attempt to redefine them.

## Pitfalls and recommendations

Since modern approaches yield too many observations for a human brain to recall simultaneously, we need to devise rules with which to program a computer to recognise clinically-important signals. For this reason, we must explicitly state the desirable characteristics of the clinical patterns we want to find. Here we state some of the pitfalls that may limit progress in this field, and make recommendations to guide future work.

### No stratification without causation

By definition, there are always subgroups within a heterogeneous population: these may be valid subgroup observations, meeting mathematical criteria for robustness and reproducibility, reliably separate from each other, and consistent in past populations and future ones. But in many cases they are clinically and biologically uninformative. What makes a clinical subgroup useful is the presence of an underlying, modifiable, causal mechanism.

**Figure 1:**
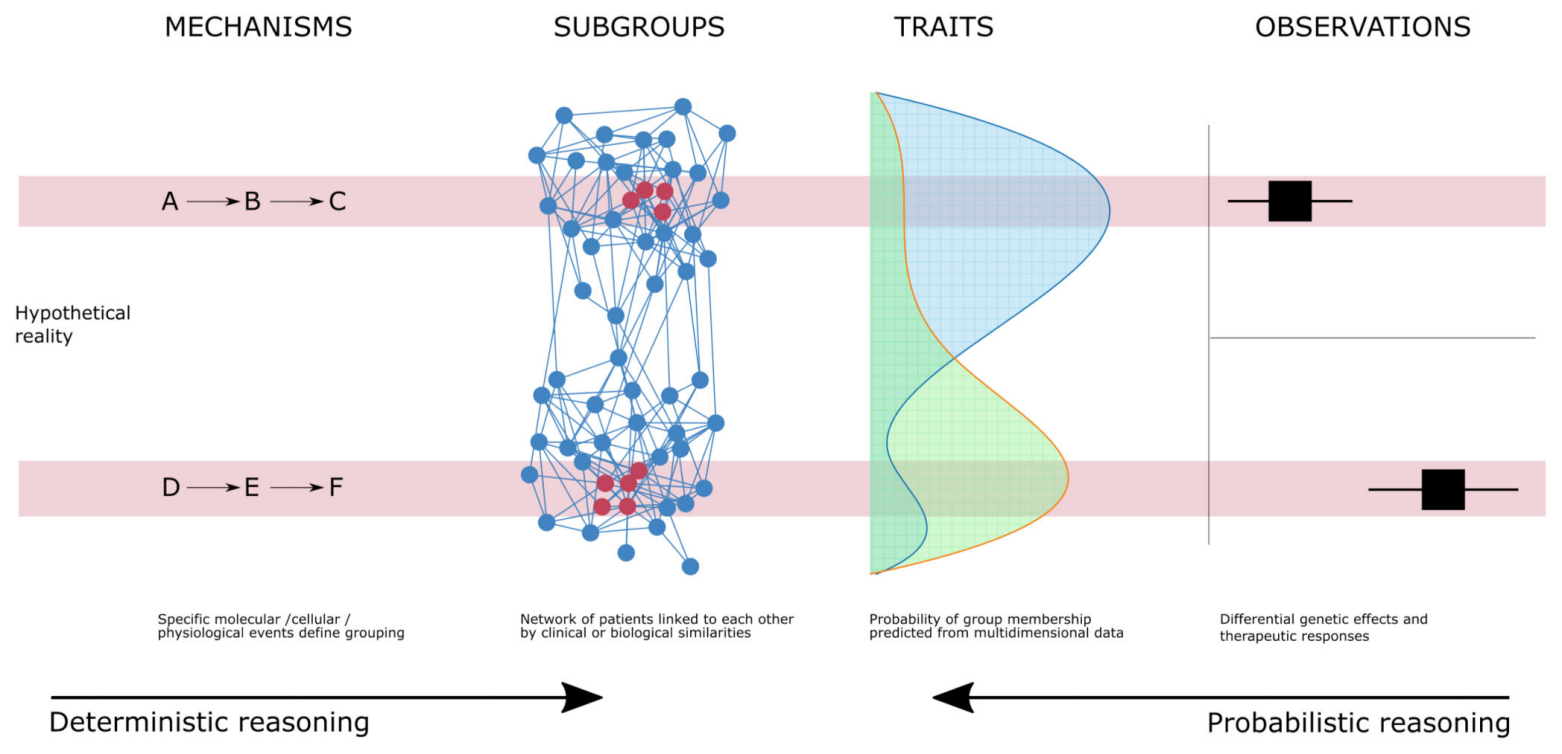
Illustration of the complementary perspectives from probabilistic or deterministic reasoning. Our observations in genetics, clinical trials and epidemiology lead to inherently probabilistic evidence (viewing from the right), but there are underlying biological or physiological events that determine disease state (progressing from the left). Patients are shown in a network grouped by similarity, with archetypal patients highlighed in red.

Robust causal inference is a primary objective of the scientific method in all cases we discuss, but different philosophical perspectives impact on the approach. The key distinction is between deterministic reasoning, which frames stratification around the assumption of an underlying, unseen, chain of causal events, and probabilistic reasoning, which views stratification as the combination of a range of semi-quantifiable probabilities of benefit or harm. Both are valid approaches; there is no dichotomy (Figure 1). In reality, both approaches are used simultaneously, overlap, and together lead to a deeper and more useful understanding of reality. A purely probabilistic approach in the absence of causal mechanisms has limited utility, because any prediction made in one population cannot be reliably translated to a future population. A deterministic approach, building stratification based on causal events, has the advantage that true causal mechanisms are predictive across populations in different circumstances, but this is very difficult (or, under a rigid definition of causality, impossible) to achieve. In reality, a tractable option is to pursue causal mechanisms as best we can, using them as a tool to improve the reliability of predictions about future populations of patients with sepsis.

**How to identify causal mechanisms driving sepsis outcomes.**

Using randomisation as the primary tool to infer causation:

- Opportunistic randomisation in genetics (Mendelian randomisation) to rapidly test many therapeutic hypotheses
  – Test specific drug targets
  – Test for mechanistic coherence within hypothetical subgroups
- Deliberate randomisation in clinical trials to definitively test carefully-selected therapeutic hypotheses Rigour in identification of sepsis subgroups with coherent causal mechanisms:
- Co-enrolment and data sharing to maximise yield from intervention studies
- Use high-quality data visualisation to incorporate domain specific knowledge from experts
- Important caveats:
  – Subgroup differences in disease outcome driven by disease severity are not proof of differences in underlying mechanism.
  – Treatable traits may not be sepsis-specific, and may occur in other syndromes.
  – Not every individual will fit into a subgroup.
  – Focus on subgroups with a high level of intra-group similarity: archetypes.
  – There may be many subgroups, however, useful subgroups are those that may benefit from specific therapies.

Trial designs:

- Use genetics to test and prioritise proposed subgroups, to improve statistical efficiency of definitive trials
- Basket trials focusing inclusion on a clinical feature that may span several different diagnoses
- “In-flight” modification of trial inclusion criteria to enable detection and testing of subgroups

Randomisation remains our primary tool to assign a causal role to any factor, because it can balance both measured and unmeasured confounding variables. Two types of randomisation offer the potential to draw robust causal inferences with quantifiable uncertainty:

1. deliberate randomisation (e.g. in clinical trials or model studies)
2. opportunistic randomisation in nature (e.g. random assignment to genotype at conception)

#### Inferring causality: deliberate randomisation in clinical trials

The most direct and interpretable causal evidence in clinical medicine comes from trials. This evidence comes with a fundamental limitation, which is common to both Bayesian and frequentist designs: any attempt at stratification makes the comparison groups smaller, drastically cutting the probability of detecting a real therapeutic signal in a subgroup, above the noise created by chance variation.

But real therapeutic signals in subgroups exist, are important, and can modify clinical practice. Detecting them requires a principled approach, because the number of possible subgroups in a trial population is so vast that it is a near certainty that some subgroups exist in which large effects for benefit or harm have been observed by chance alone. In order to overcome this, trials define a carefully limited number of subgroups for secondary analyses *a priori*. This approach can work: for example, the RECOVERY trial in the UK discovered a therapeutic benefit from steroid treatment in some patients with Covid-19 (those with hypoxaemic lung injury), and a trend towards harm in others (hospitalised patients not requiring oxygen therapy).^27^ This subgroup analysis was defined in advance, and the differential treatment effect was very strong. Perhaps for these reasons, and because of the unusual circumstances of the pandemic, this result led to an immediate change in clinical practice.^28^

Efficiency is the main problem in deriving causally coherent subgroups from trial data. The number of subgroups that can be tested, and the number of treatments that can be tested simultaneously, are limited. These limitations are baked in: if a clinical trial continued long after the treatment had been shown to be effective, it is very likely that differential treatment effects between subgroups would be identified, since the relative benefit and harm varies between patients (a concept referred to as heterogeneity of treatment effect, HTE). But no trial would ever continue to allocate patients to miss out on a treatment that has been shown to be effective. The ethical imperative in trial design acts to limit the power to detect subgroups. This limitation does not apply to another method to draw causal inferences about biological mechanisms - host genetics - in which “spare” statistical power is generated routinely.

#### Inferring causality: opportunistic randomisation in genetics

Since some genetic effect sizes can be very large, and because the same hypothesis is tested repeatedly in each new study of a given disease, aggregated genetic signals are often overpowered. For example in the largest study of susceptibility to critical illness in Covid-19, the strongest association has a P-value less than 1x10^-253^.^29^ Unlike clinical trials, genetic studies do not stop detecting a signal after it reaches criteria for significance. This means that an extensive reservoir of spare discovery power is generated, and can be divided among subgroups to *test* stratification hypotheses (Figure 1).

Genetics has the potential to cut through the intractable complexity of the human immune system to identify levers that alter outcome.^7^ For example, genes that associated with cardiovascular disease and cholesterol levels encode proteins targeted by drugs. In the case of PCSK9 inhibitors, a new effective drug was discovered from a host genetic study. Similarly, for the first time in critical care medicine or infectious diseases, a host genetic signal near the *TYK2* gene^30^ led directly to the discovery of a new, effective treatment for Covid-19: baricitinib.^31^ This drug was one of 9 candidates under consideration for inclusion in the RECOVERY trial, and like others on the shortlist,^32^ had some support from bioinformatics predictions and small trials.^33^ The genetic signal influenced the prioritisation of baricitinib ahead of other potential treatments (personal communication, Patrick Chinnery, Chair, UK CTAP^34^).

The primary methodology used for causal inference in genetics is a special case of instrumental variable analysis called Mendelian randomisation. Briefly, at conception, each individual is assigned to a given genotype at each position in their genome. These genotypes are selected at random when gametes are formed through meiosis. Since the genome is also largely static from conception throughout life, this creates a powerful tool for asking causal questions about biological mediators. For example, where the same genetic variants are associated with mediator levels (i.e., where a given genotype changes the amount of a mediator that an individual produces) and with a clinical phenotype (e.g. the development of a disease), they can be used to infer causal relationships between mediators and clinical phenotypes.^35^ The analysis treats patients as if they had been randomised, in a trial, to receive higher or lower amounts of the mediator, and asks, what effect does this have on development of the disease? The approach relies on several explicit assumptions, of which the most important is the assumption that there is not an alternative mechanistic pathway linking a genetic variant to a clinical outcome.^36^ This assumption is often violated, since genetic variants tend to have multiple effects (“pleitropy”),^37^ and infinitesimally small genetic effects on other traits are widespread (and possibly ubiquitous).^38^

**Figure 2:**
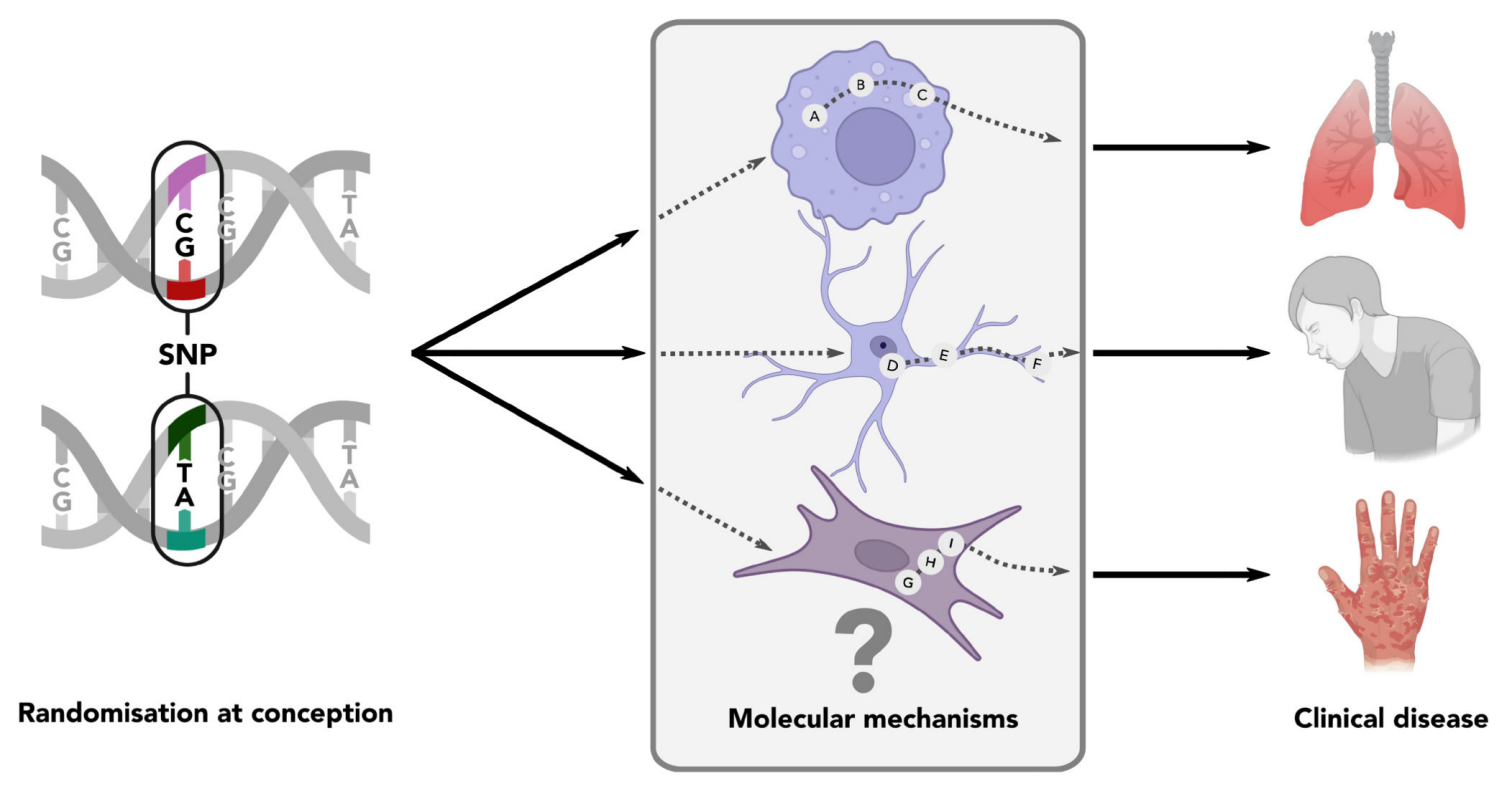
Role of molecular QTL (including eQTL) in filling in the gaps in mechanistic understanding of genetic mechanisms of disease.

Mendelian randomisation can in theory be used to test hypotheses about a causal role for any exposure variable that can come under strong genetic influence. The most directly relevant exposures for mechanistic inference are genetic variants (quantitative trait loci, QTL) that change the amount of a given molecule that is produced in a particular biological context (e.g. RNA expression (eQTL) or protein concentration (pQTL)). Where these molecular QTL are also associated with a clinical outcome, they provide an opportunity to fill the mechanistic gaps between genetic variants and disease states (Figure 2). Disease-associated molecular QTL relationships are highly cell-type and -state dependent, so despite a rapid recent expansion in known molecular QTL^39,40^ it is likely that many important effects remain to be discovered.

**Figure 3:**
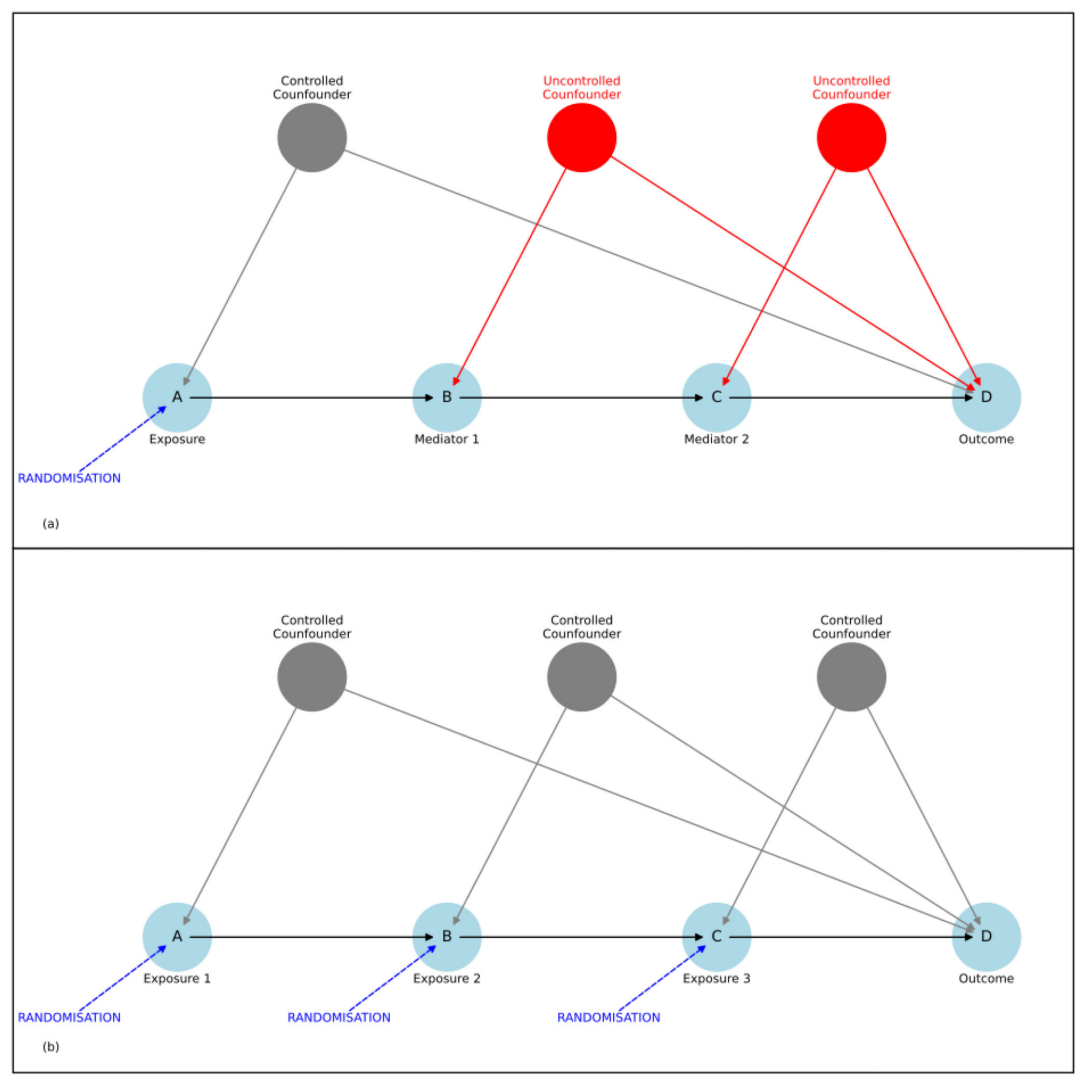
Causal models of a hypothetical pathway in which three molecules (A, B, and C) form a pathway that leads to a potential clinical outcome (D). (a) In an experiment, patients can be randomised to receive an inhibitor of ‘A’. Randomisation balances confounders that affect the relationship between exposure and outcome, enabling robust causal inference. But randomisation does not balance the effect confounders, so the causal contributions of intermediate mediators cannot be directly inferred. (b) A proposed composite of multiple experiments in which each mediator is tested as a separate exposure. Using Mendelian randomisation, if there are known genetic effects (eQTL) on each mediator, then each element in the pathway can be efficiently tested, using randomisation at conception to balance confounders.

With advancing knowledge of molecular QTL, genetics provides the potential to efficiently test multiple causal hypotheses about mediators in the same pathway in a composite design, by controlling for confounders at each step using Mendelian randomisation (Figure 3).[10.1534/genetics.117.300191] The same approach would be technically possible, but inefficient and costly, using a series of targeted interventions in randomised controlled trials.

The prediction of therapeutic effects from genetics depends on a simple parallel: in some circumstances, the effect of a genetic variant mimics the effect of a drug. This arises where the genetic variant modifies molecular mechanisms in a similar way to a given treatment. A further parallel is expected to exist with heterogeneity of treatment effect. Just as individual patients have different probability of net benefit from a given drug treatment, there is heterogeneity of genetic effect across a population of patients. Taking the example of corticosteroid treatment in Covid-19, the population in which greatest therapeutic benefit was seen, is also the population in which the strongest genetic signals for association with hypoxaemic respiratory failure are seen.^29^

Importantly, the proportion of variance in a common clinical outcome explained by a single genetic variant is usually very low, which leads to a common misconception that the signals are not useful. In fact, this is an expected finding in complex trait genetics and does not preclude the use of genetics in identifying disease mechanisms.

We believe that the time has come to obtain and collate genotype data from tens of thousands of patients with sepsis. Global, “open-source”^41^ efforts such as GenOMICC (Genetics Of Mortality In Critical Care) offer the potential to significantly advance biological understanding of sepsis and other forms of critical illness.

### How to identify subgroups and traits

#### There is no reason why the biggest stratification signal should be the most informative

A simple example reveals the challenge. In many cities around the world, the population divides their allegiance between two or more sports teams. Classifying the patients presenting to a local hospital in such a city according to the colour of their clothing would result in robust groupings of patients that meet the most stringent mathematical criteria. Demographic and behavioural differences between these groups may result in different disease prevalence, biological characteristics and even clinical outcomes. This may correlate strongly with other factors, including socioeconomic status, environmental exposures, and even religious beliefs. But these traits are highly unlikely to reveal therapeutically-actionable underlying mechanisms. The largest stratification is the easiest to detect, but a smaller signal with a coherent causal mechanism could be much more important.

#### Conflating severity with mechanism

Since sepsis is a response syndrome, differential severity and differential disease mechanisms are often indistinguishable. In critically ill populations, the probability of death varies continuously over a wide range. This has a direct mathematical effect on the probability of net benefit from therapeutic interventions, and hence on the observed treatment effect in clinical trials. This form of heterogeneity of treatment effect means that there are always some patients with a much higher chance of net benefit from an effective therapy, and conversely, some patients with a higher probability of null effect, or harm.^42^ The impact of HTE is that the optimal probability of net benefit from a given therapy is a balance between the benefit and harm associated with that therapy, and is hence specific to that therapy.

Similarly, different patients prioritise different disease outcomes: some may value survival above all else, whereas others will place greater value on symptom control. So the choice of the ideal group of patients to receive a given therapy is not only determined by the probability of net benefit, but by the definition of “benefit” for each patient. Disease severity has direct and important therapeutic consequences, but it is conceptually distinct from disease mechanism. Heterogeneity of severity acts on a single dimension, and is quantifiable by definition for any clinical outcome. In contrast, mechanistic heterogeneity is as broad as our current understanding of disease biology, and as multidimensional as the number of different pathophysiological processes that exist. Since mechanistic heterogeneity is expected to overlap with severity heterogeneity, we recommend that a difference between subgroups in disease outcome should not be taken as evidence of difference in underlying mechanism.

#### The spotlight fallacy

**Figure 4:**
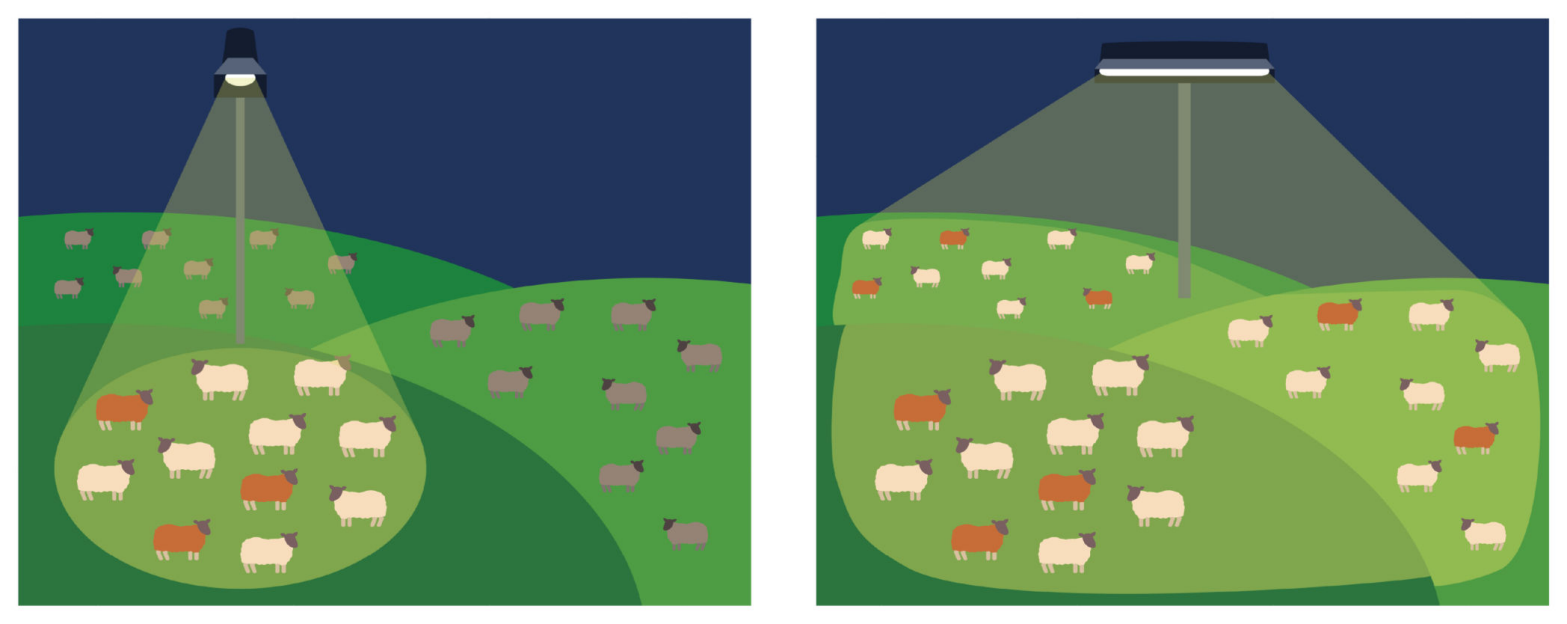
The spotlight fallacy. An observer looking at a flock of sheep in the darkness cannot see what colour each sheep is. Shining a spotlight on one field reveals a hidden subgroup of brown sheep among a flock of white ones (a). The observer falsely concludes that the brown sheep are only to be found in one field, but in fact they can be found in other fields if they are illuminated (b).

This form of selection bias affects the interpretation of any study using new technology to examine a group. Subgroups of a set are interpreted to be specific features of that set, neglecting the possibility that the same subgroupings apply equally to other sets (Figure 4). For example, imagine a new technology is used to observe patients meeting a certain condition, such as sepsis (as if a new kind of spotlight is being shone on this group). Subgroups are found within these patients. This observation is then interpreted to be a new discovery about patients with sepsis. But it may well be that the same observation can be made about patients with many other clinical problems, but since no-one has ever looked for the same observations in other conditions, this has been overlooked.

Real mechanisms of disease do not always restrict themselves to clinically-obvious syndromes. For this reason, the definition of treatable traits is *syndrome-agnostic*. This is self-evident from numerous examples in medicine - to take a trivial example, shock is modifiable by noradrenaline, but can occur in a broad range of syndromes. It is not a surprise that subgrouping signals are detected in multidimensional data analysis. Probably the first example of this is the observation that the same subgroups can be detected in patients with ARDS^43^ and mild-moderate pancreatitis,^11^ suggesting that the subclassification signal in ARDS is generalisable to a broader range of syndromes.

#### It is not necessary to assign every patient to a subgroup

Diagnoses are sometimes made with a high degree of confidence and sometimes with considerable uncertainty. It is common, particularly in acute presentations, for patients to have no diagnosis at all. Studies looking to discover new diseases within heterogeneous syndromes should employ the same humility as a clinician at the bedside, accepting that in many cases, we aren’t sure which category fits best. Subgrouping studies in sepsis have often assigned group/cluster/class membership to every patient within the study population.

The converse of the spotlight fallacy is the assumption that, within a given group of patients, every individual must be classifiable. Whilst we take it as read that every patient is deserving of care, it is not true that every patient can be assigned to an informative subgroup. When we subdivide a group of patients with the same syndrome, we must have the humility to ask, what if some patients don’t really have that syndrome? Or what if the definition of the syndrome itself is incoherent? If either of these conditions are true, some patients will not belong in any subgroups of that syndrome.

The consequences of this depend on the mathematical approach to classification. Many approaches derive from general classification methods and begin with the assumption that all entities must be classified. Probabilistic methods, which derive a probability of class assignment for each individual, can overcome this limitation, but depend on the user to avoid this pitfall when interpreting the results. Some patients are not classifiable, and some subgroups are not informative.

Uninformative subgroups are not a new problem, and the solution adopted by our medical ancestors presents a tractable route forward: archetypes. Archetypes are familiar to clinicians worldwide as those rare, uncomplicated presentations of a single disease. They are known variously as “classical”, “typical” or “canonical” presentations of disease, and taught to medical students primarily as a tool to preserve memory. The concept may also help to protect us from some of the pitfalls listed above: by focusing only on what is interpretable and informative, we can avoid the difficulties created by attempting to create a single solution for a complex and heterogeneous clinical population. Focusing on subgroups with a high level of intra-group similarity is more likely to yield subgroups across which the underlying causal mechanisms of disease are demonstrably shared (Figure 1).

#### The number of subgroups does not matter

Patients do not fall into discrete, mutually-exclusive subgroups. The presence or absence of disease is not a binary classification. This is evident because:

- patients often present with many overlapping diagnoses simultaneously
- diagnostic observations often have a high degree of measurement uncertainty
- most disease processes exist on a wide spectrum of severity, which may vary over time and alters the balance between risk and benefit for any given treatment

Two conclusions follow: firstly, an optimised subgroup definition for therapeutic benefit will be specific to a given therapy, and may not generalise to other therapies, particularly those that target a different mechanism. Secondly, the number of subgroups observed within a group of patients is wholly determined by the application of arbitrary thresholds along a range of continuous variables. Hence, the absolute number of subgroups is uninformative. What matters is the number of *useful* subgroups. For example, a study of sepsis that observed that a group of patients with a particular combination of test results (e.g. SARS CoV-2 antigen and hypoxaemia) would benefit from treatment with steroids, IL-6 inhibition, and baricitinib, would be a major step forward, even if the same study also identified other, uninformative, subgroups.

#### Consider the pathogen

It is already true that a single class of laboratory tests can stratify patients with sepsis according to immediately therapeutically-actionable clinical features: pathogen diagnostics reveal a target for antimicrobials, and increasingly, for immunomodulatory therapy to prevent organ injury.^27,31,44^ As molecular diagnostics improve and generate increasingly complex information about potential pathogens,^45^ it will be important to integrate microbial diagnostics into analyses of multidimensional host observations.

#### Domain-specific knowledge

Analysis of deep phenotyping does not need to start from a position of total ignorance. Biological processes in innate immunity are some of the most-studied in any species. A fundamental challenge is incorporating domain-specific knowledge into analytic strategies to detect treatable traits. This requires two-way communication. There is a clear unmet need, first to encode the existing knowledge of clinicians and biologists for use in machine learning, and secondly to visualise the richness of data from deep phenotyping such that it can be interpreted by clinicians.

#### Acquisition of deep phenotyping data

A significant practical challenge exists in linking the tools described above for causal inference with the deep biological data that can reveal stratification signals. Obtaining any additional data or sample in a clinical trial or host genetic study comes at a cost by depleting the financial, human or institutional capacity available to recruit new patients. We propose that, since it is not necessary to obtain deep phenotype data from every patient in a study, co-enrolment and data sharing among trials, genetic and phenotyping studies will substantially accelerate progress in sepsis research and mechanisms to encourage this should be incentivised by both funders and research groups.

### Trial designs

#### Basket trials and predictive enrichment

There are multiple examples in critical care of “basket” trials^46–48^ using syndrome-agnostic, biomarker-defined inclusion criteria (i.e. testing whether these traits are treatable). The concept has been very effective in oncology (reviewed here^49^). The insight in basket trials is, fundamentally, to avoid the spotlight fallacy (Figure 4) by including similar patients from across multiple different disease states or syndromes. For example the ongoing TRAITS trial in Scotland (https://traits-trial.ed.ac.uk/) recruits critically ill patients with shock and raised CRP for randomisation to imatinib, or those with lymphopaenia and respiratory dysfunction for randomisation to budesonide/baricitinib. This is a true “basket” design in which the inclusion criteria are not restricted to a clinical diagnosis or “spotlight”.

Predictive enrichment is the selection of patients who are more likely to respond to a given therapy (compared with the similarly-named prognostic enrichment, which is the selection of patients at higher risk of an outcome of interest).^50^ An early example of this logic is the 2004 MONARCS trial, which employed a rapid test in severe sepsis patients to identify a subgroup with high circulating IL-6, whilst randomising all patients to receive an anti-TNF monoclonal antibody.^51^ More recently, the EUPHRATES trial of polymixin B haemoperfusion to remove endotoxin enriched for patients with high endotoxin levels,^52^ tying the biomarker used for patient selection to the proposed causal mechanism of the intervention. A broad definition of predictive enrichment would include stratification by site of infection^53^ or by specific organ failure.^54^ If the mechanistic rationale for enrichment is correct, this approach increases the probability of detecting a true effect, at the cost of decreasing generalisability.

#### “In-flight” precision trials

Where there is residual uncertainty about therapeutic effect in a subgroup, after the primary analysis of a trial is complete, there is a strong argument for continuing to randomise within that subgroup. We propose that subgroup detection “in-flight” in clinical trials could both rescue effective treatments that would otherwise be rejected, and protect patients from receiving treatments that are more likely to cause them harm. Funding bodies should facilitate extended refinement of therapeutic indications by supporting ongoing recruitment within subgroups identified through a principled analysis. Such subgroups may be identified *a priori* or through unsupervised analyses of trial data, since ultimately the therapeutic effect in any chosen subgroup will be subjected to a rigorous test through subsequent randomisation.

## Conclusion

Many of these pitfalls can almost always be avoided by a simple, intuitive approach which is too often missed in large scale studies: maintaining a focus on the desirable real-life destination of subclassification research. By considering the ultimate goal - to delineate a landscape of informative disease archetypes - the ultimate utility of sepsis research can be significantly improved.

Like SIRS, sepsis is, fundamentally, more of a hypothesis than a disease. Perhaps our mistake has been to use the term in the context usually reserved for diseases - “treatment of sepsis”, “diagnostic criteria for sepsis” - rather than the more circumspect language used to describe a theory, that is, that organ damage is caused by a common pathway of host immune mechanisms precipitated by a broad range of infectious triggers. We contend that progress in sepsis research will require, above all, a focus on causal mechanisms driving clinical outcomes.

There is no single objectively-true view of the landscape of human disease. We consider the defining feature of a successful view of the landscape to be *utility*. Subgrouping aims to identify mechanistically coherent disease archetypes with value beyond the clinically obvious, with differential genetic predisposition and treatment response. Ultimately, we need to move beyond classification, to recognising, explaining and modifying continuous traits within the sepsis population.

## Methods

The objectives of the ISF Colloquium were to: (a) engage experts from data analysis, machine learning, systems biology and related disciplines in the clinical problem of sepsis and how to identify and treat the underlying biological processes; (b) Establish effective communication between domain experts in clinical science, immunology, genomics and data science; and, (c) identify and overcome the barriers to achieving these goals in sepsis.

The International Sepsis Forum Executive Committee (M.S., T.C., S.F., and Elaine Rinicker) appointed co-chairs for the colloquium (J.K.B & A.R.), who identified contributors based on expertise relevant to the topic. A deliberate attempt was made to engage experts form outside of critical care medicine and infectious disease research. Experts in systems biology, high-dimensionality data analysis, genetics and genomics, transcriptomics, clinical epidemiology were asked to participate. Through an iterative process of snowball sampling by recommendation, a highly-engaged set of experts was gathered for the two-day colloquium, which occurred 6 weeks before the first cases of Covid-19 were reported, and provided the opportunity to test many of the ideas proposed. The final report summarises a consensus among the authors.

## Take Home Message

Until now, causal inference has been lacking from efforts to tackle heterogeneity in sepsis, ARDS and other forms of critical illness. The ability to test for causality using genetics, trial data, and other methods provide a tool to resolve this, and ultimately will enable us to find what we all want: mechanistically-coherent archetypes of critical illness.
